# Awareness and Attitudes Toward Artificial Intelligence Language Generation Models in Medical Education: A Cross-Sectional Questionnaire Study Among Medical Students in Southern China

**DOI:** 10.7759/cureus.89425

**Published:** 2025-08-05

**Authors:** Min Zhang, Tao Liu, Xiang Peng, Yuanhan Chen, Min Zhi

**Affiliations:** 1 Department of Gastroenterology, Sixth Affiliated Hospital, Sun Yat-sen University, Guangzhou, CHN; 2 Biomedical Innovation Center, Sixth Affiliated Hospital, Sun Yat-sen University, Guangzhou, CHN; 3 Division of Nephrology, Guangdong Provincial People's Hospital (Guangdong Academy of Medical Sciences) Southern Medical University, Guangzhou, CHN; 4 Department of Internal Medicine, Second Ward, Nyingchi People's Hospital, Nyingchi, CHN

**Keywords:** artificial intelligence language generation models, attitude, feasibility study, medical education, medical students, questionnaire survey

## Abstract

Purpose

To evaluate the feasibility of artificial intelligence language generation models (AILMs) in medical education, we examined the utilization patterns and attitudes of medical students in a developed area of Southern China.

Methods

We conducted a cross-sectional questionnaire survey assessing educational background, awareness, usage, and attitudes towards AILMs. Attitudes were measured using a five-point Likert scale, where scores of 4 or above indicated support, scores of 2 or below indicated opposition, and a score of 3 indicated a neutral stance.

Results

Among the 254 respondents, the average awareness score for AILMs was 2.4. AILMs were primarily used for solving medical and academic problems. Although students were aware of many domestic AILM products, foreign products were preferred. More than half of the students used AILMs less than once a week, and 13 (5.1%) students had never used them.

A significant portion supported the integration of AILMs in current (187/254, 73.6%) and future (194/249, 78.0%) education, with a strong correlation between these attitudes (*χ*² = 46.351, P < 0.001). Concerns about technological immaturity were a major reason for opposition. A higher proportion of those who opposed the use of AILM had advanced computer skills compared to those with lack of or basic computer skills (10/47, 13.5% vs. 9/177, 5.1%, P = 0.010). Even after adjusting for specialty and academic performance, advanced computer skills were independently linked to opposition (OR 2.959, 95% CI 1.109 - 7.898).

Conclusion

While medical students generally support the use of AILMs, broader acceptance requires addressing challenges such as enhancing the quality and promotion of domestic AILMs and considering the diverse perspectives of individuals with varying levels of computer proficiency.

## Introduction

Computer-based medical consultations were proposed in the past to assist in the diagnosis, treatment, management, and prediction of diseases [1,2]. However, the limited prevalence of computers and technological constraints, such as low computational power and insufficient data storage, hindered the widespread adoption of artificial intelligence (AI) in medical education at that time [3].

In recent years, the widespread availability of computers and rapid advancements in AI technology, particularly the development of artificial intelligence language generation models (AILMs), have opened new avenues for integrating AI into medical education [4-6]. These AILMs can personalize learning experiences, serve as intelligent tutors to answer questions, automate assessments and feedback, and generate a wealth of educational resources. This integration promotes innovative thinking and interdisciplinary learning, ultimately enhancing the quality of education. As AI continues to permeate medicine, its presence in medical education is becoming increasingly evident. However, despite AILM's potential to revolutionize teaching methods, the computer literacy and acceptance levels of medical students towards these emerging technologies remain unclear.

Recent surveys have revealed that medical students' awareness of AI is not as high as expected. For instance, while nearly half of American medical students employ ChatGPT (OpenAI, San Francisco, CA, USA) for study tasks, 31% seldom use it [7]. In the UK, a significant 92% of medical residents reported a lack of AI training in their curriculum and expressed a need for such education [8]. Similarly, in the Balkans, only 38.2% of medical students reported familiarity with AI concepts, although 44.8% anticipated using AI in their future practice [9]. Medical students and residents in Egypt generally hold a negative attitude towards the integration of AI technology in healthcare [10]. Among Thai radiology students, less than one-third felt they had a basic understanding of AI [11]. Indian medical students frequently voice ethical concerns, with nearly 75% indicating a need for training in medical AI [12]. Additionally, about three-quarters of Pakistani medical students have not received any formal AI education before or during their studies [13]. These findings highlight substantial challenges in the integration of AI into medical education at this stage.

This cross-sectional study aims to investigate the feasibility of integrating AILMs into medical education in China and other regions with similar conditions by examining the background knowledge and attitudes of medical students in Guangzhou, a representative developed city in China. The findings are intended to provide empirical evidence for the future implementation of AILMs in medical education and offer insights for other regions.

## Materials and methods

Questionnaire design

The self-developed questionnaire was designed to comprehensively assess the educational background, awareness, usage patterns, and attitudes of medical students towards AILMs. It consisted of 15 key questions, primarily in multiple-choice format, to ensure ease of completion and minimize respondent fatigue. The questions were organized into four main sections: 

Educational Background

This section collected information on the respondents' current academic standing, specialty, and self-reported academic performance. Respondents' computer proficiency was categorized into three groups: Basic (minimal or no computer skills, or ability to perform basic tasks such as browsing the web, sending emails, and using social media), Intermediate (proficiency in office software like the Microsoft Office suite, data analysis, and presentation creation), and Advanced (programming skills and ability to edit images, audio, and video).

Awareness and Usage of AILMs

Respondents were asked about their familiarity with various AILMs, both domestic and foreign, and their frequency of use. Additionally, they were required to specify the purposes for which they utilized these models.

Attitudes Towards AILMs in Medical Education

This section employed a five-point Likert scale to quantify students' attitudes towards the integration of AILMs in medical education. The scale ranges from 5 to 1, representing Strongly agree, Agree, Neutral, Disagree, and Strongly disagree, respectively [14]. Scores of 4 or above were defined as supportive attitudes, while scores of 2 or below indicated opposition. Neutral responses (score of 3) were considered non-supportive. Thus, this study separately analyzed non-supportive (including neutral and opposing) and opposing attitudes. To enhance reliability, an additional open-ended question was included to gather qualitative feedback on the perceived advantages and disadvantages of using AILMs in future medical education.

Preferred Training Formats

Respondents were asked about their preferred formats for AILM training, including the duration and type of training sessions.

To ensure the clarity and reliability of the questionnaire, several measures were taken during its design. Firstly, anonymity was guaranteed to encourage honest responses. Secondly, questions were refined through group discussions involving two senior medical educators and five medical students with varying levels of computer proficiency to eliminate potential ambiguities. Thirdly, the questionnaire was simplified by omitting redundant options (e.g., age, given the uniformity of respondents' age range). Additionally, the questionnaire underwent expert review to ensure content validity and was pilot-tested to assess face validity, ensuring that the questions were clear and comprehensible. The final version of the questionnaire was pre-tested to ensure it could be completed within 40-90 seconds under continuous filling conditions. The survey was conducted using an online questionnaire platform (Wenjuan), which is accessible via both computers and mobile phones. The questionnaire is provided in the Appendix.

Ethical approval and consent

The study was reviewed and approved by the Ethics Committee of the Sixth Affiliated Hospital of Sun Yat-sen University (Approval No.: 2025ZSLYEC-205). All participants provided informed consent through the online questionnaire platform, agreeing to participate in the study and provide the relevant data. We clearly stated the purpose of the study, how the data would be used, and the rights of the participants at the beginning of the questionnaire to ensure that all participants were fully informed and participated voluntarily.

Data collection

The survey was conducted from October 23 to October 29, 2024, targeting current third-year medical undergraduate students at Sun Yat-sen University, one of the leading comprehensive universities in Southern China.

Given that all of the targeted medical students were active WeChat users, the survey link was distributed via the WeChat social media platform to ensure wide reach and accessibility. A total of 263 responses were initially collected, and after excluding nine responses with completion times less than 40 seconds (indicating potential insincerity), 254 valid responses were included in the final analysis.

Analysis

Data were exported from the online survey platform and analyzed using SPSS software (IBM Corp., Armonk, NY, USA). Descriptive statistics were used to summarize the respondents' characteristics, awareness, and usage patterns of AILMs. Correlation analysis was performed to assess the relationship between current and future attitudes towards AILM integration. Additionally, univariate and multivariate logistic regression analyses were conducted to identify factors associated with opposition to the application of AILMs in medical education. For comparisons among groups, *χ*^2^ tests were used. When significant differences were found among multiple groups, pairwise comparisons were conducted with the significance level (*ɑ*) adjusted using the Bonferroni correction (*ɑ* = 0.05/3 = 0.0167). The overall significance level for all statistical tests was set at *P* < 0.05, with adjustments made where necessary for multiple comparisons.

## Results

**Figure 1:**
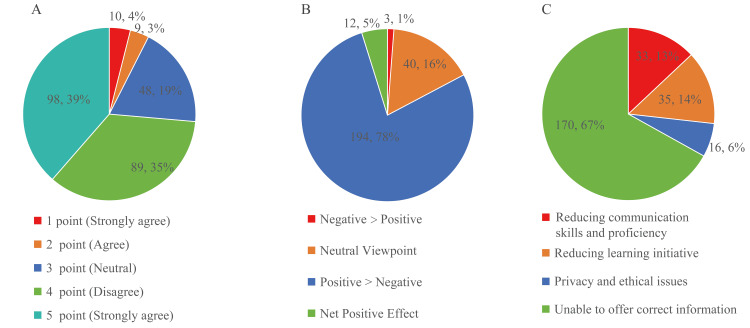
Attitudes on AILM Integration in Education A. Attitudes towards the introduction of AILM in medical education at the current stage. A five-point Likert scale is used to measure quantitative attitudes, with higher scores indicating more supportive attitudes. B. Attitudes towards the future introduction of AILM. Note: Five respondents stated that they were unable to confirm their future attitudes, thus B represents the attitudes of 249 respondents. C. Major concern about the application of AILM in medical education AILM: Artificial Intelligence Language Generation Model

A total of 263 current medical undergraduate students completed the survey. After excluding nine responses with completion times less than 40 seconds, 254 responses were included for final analysis. The majority of respondents supported the introduction of AILM in teaching. Based on the five-point Likert scale, 187 (73.6%) participants scored 4 or higher, and 48 (18.9%) scored 3, indicating a neutral stance, but there were also 19 (7.4%) individuals who expressed opposition (Figure 1A, see Table 1 for detailed breakdown).

**Table 1:** Basic information based on different attitudes Pairwise comparisons: ^a^ Supportive vs. Opposing (*χ*^²^ = 3.856, *P* = 0.050, not significant after Bonferroni correction); ^b^ Neutral vs. Opposing (*χ*^²^ = 6.573, *P* = 0.010, ^*^significant after Bonferroni correction). AILM: Artificial Intelligence Language Generation Model

	Supportive attitudes	Neutral attitudes	Opposing attitudes	*χ*^2^ value	*P* value
Sex				0.257	0.879
Male	80 (72.1%)	22 (19.8%)	9 (8.1%)		
Female	107 (74.8%)	26 (18.2%)	10 (7.0%)		
Specialty				0.945	0.624
Clinical	146 (73.0%)	40 (20.0%)	14 (7.0%)		
Non-clinical	41 (75.9%)	8 (14.8%)	5 (9.3%)		
Academic performance				1.010	0.908
Top third	75 (76.5%)	16 (16.3%)	7 (7.1%)		
Middle third	82 (72.6%)	23 (20.4%)	8 (7.1%)		
Bottom third	30 (69.8%)	9 (20.9%)	4 (9.3%)		
Computer skill				6.525	0.038
Basic or intermediate	130 (73.4%)	38 (21.5%)	9 (5.1%)		
Advanced	57 (73.0%) ^a^	10 (13.5%) ^b, *^	10 (13.5%)		
AILM usage				0.479	0.787
No	31 (75.6%)	8 (19.5%)	2 (4.9)		
Yes	156 (73.2%)	40 (18.8%)	17 (8.0%)		

Self-reported computer proficiency

According to the classification defined in the Methods section, a total of 177 respondents (69.68%) reported Basic computer skills, 69 respondents (27.16%) indicated Intermediate skills, and eight respondents (3.15%) reported Advanced skills.

Awareness and application of AILM

**Figure 2:**
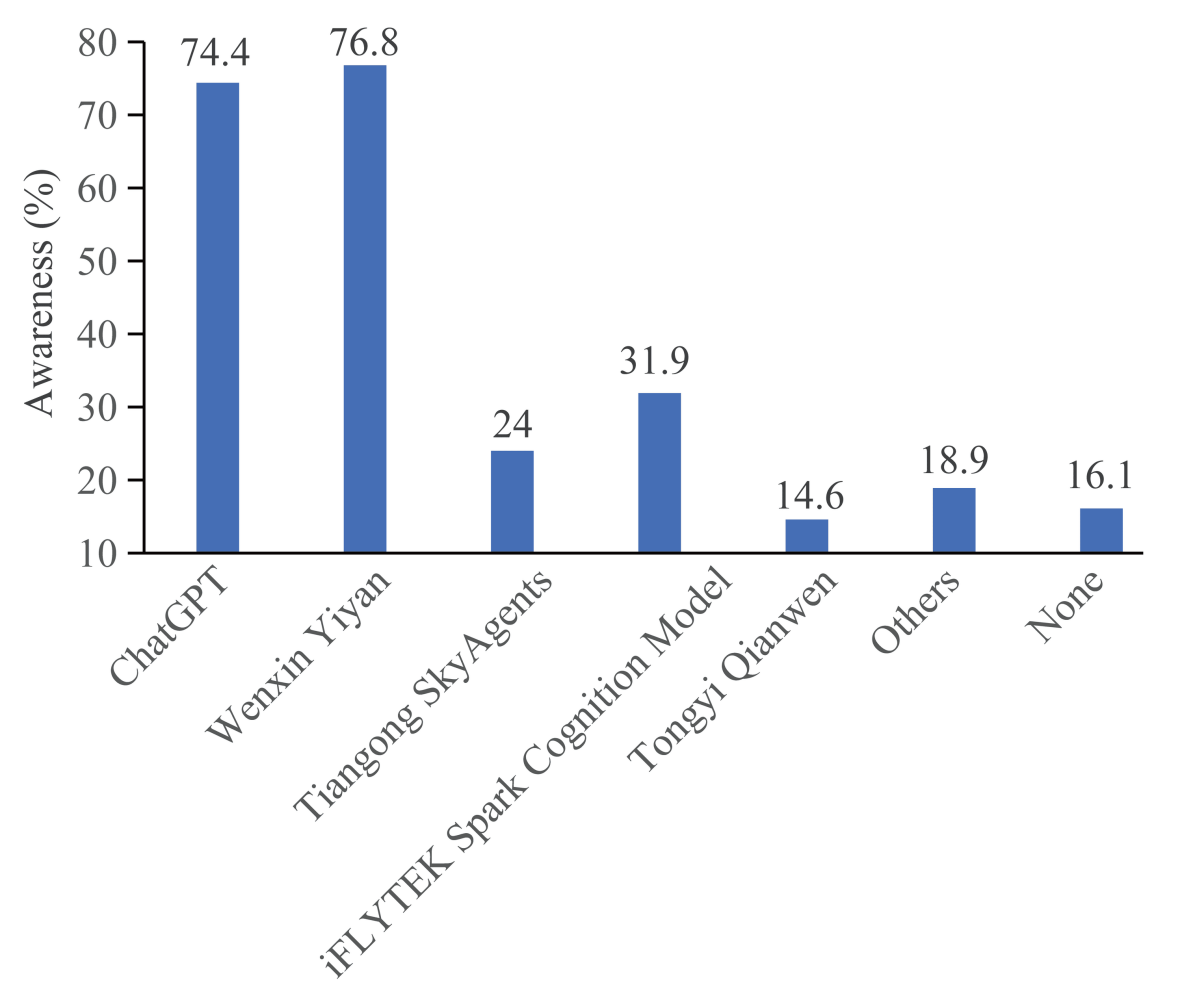
Awareness of Artificial Intelligence Language Generation Models Bar chart showing the awareness percentage of AILMs among medical students. AILM: Artificial Intelligence Language Generation Model

**Figure 3:**
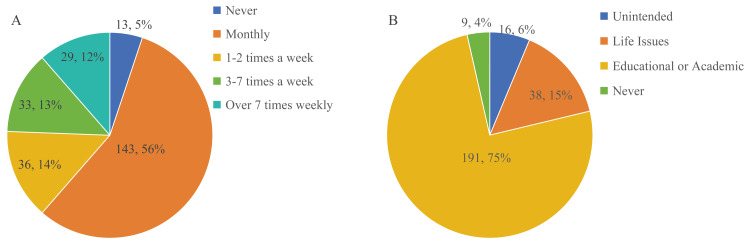
Application of Artificial Intelligence Language Generation Model A. Frequency of AILM usage among medical students. B. Purposes of AILM usage among medical students AILM: Artificial Intelligence Language Generation Model

Among the 254 respondents, there was an average awareness of 2.4 AILMs per person (median: 2.0), including ChatGPT. Besides this first AILM introduced in China, the awareness rates for other AILMs, from highest to lowest, were Wenxin Yiyan (Baidu, Beijing, China), iFLYTEK's Spark Cognition Model (Hefei, China), Tiangong AI (Kunlun Tech, Beijing, China), and Tongyi Qianwen (Alibaba Cloud, Hangzhou, China). Although the respondents were aware of a significant number of domestic AILM products, their usage tended to favor foreign products, with a preference ratio of 156:98 for foreign to domestic products. Forty-one (16.1%) individuals reported being unsure about AILM categories (Figure 2). The moderate level of awareness of AILMs suggests that there is still a need for increased exposure and education about their potential benefits in medical education, and the preference for foreign AILMs over domestic ones underscores the need for improving the quality and promotion of domestic AILM products to better meet the educational needs of medical students. Despite a relatively high awareness, most surveyed medical students did not frequently use AILMs. Over half of the respondents used AILMs less than once a week, and 13 (5.1%) individuals had never used them (Figure 3A). This usage pattern suggests that many respondents may not fully integrate these tools into their regular study routines, primarily because they perceive AILMs as useful for solving specific medical and academic problems rather than for general use. This is further supported by the finding that the primary reasons for using AILMs were to address problems related to medical course studies and professional or academic issues (Figure 3B).

Medical students' attitudes on AILM integration in education

The majority of students support the introduction of AILM in medical education at the current stage (Figure 1A). Regarding whether AILM will be beneficial to medical education in the future, attitudes are similar to those regarding its current introduction, with the majority (206/249, 82.7%) of students still believing that the benefits outweigh the drawbacks. While the overall outlook is positive, it is important to note that a significant number of students still hold neutral (40/249, 16.0%) or opposing (3/249, 1.2%) attitudes towards the future application of AILMs (Figure 1B). The respondents' supportive attitudes towards the future application of AILM in medical education are highly correlated with their current attitudes towards introducing AILM (*χ*^²^ value 46.351, *P* < 0.001). Taken together, the students' current positive attitudes are likely to persist in the future. In other words, although most students are optimistic about the current and future use of AILMs in medical education, a notable portion still maintain neutral or opposing views.

The most common concern about the use of AILM in medical courses is the worry about “AI hallucination,” where the technology may generate plausible but inaccurate information (Figure 1C). This highlights that the immaturity of AILM technology is a major reason limiting its use by medical students.

**Figure 4:**
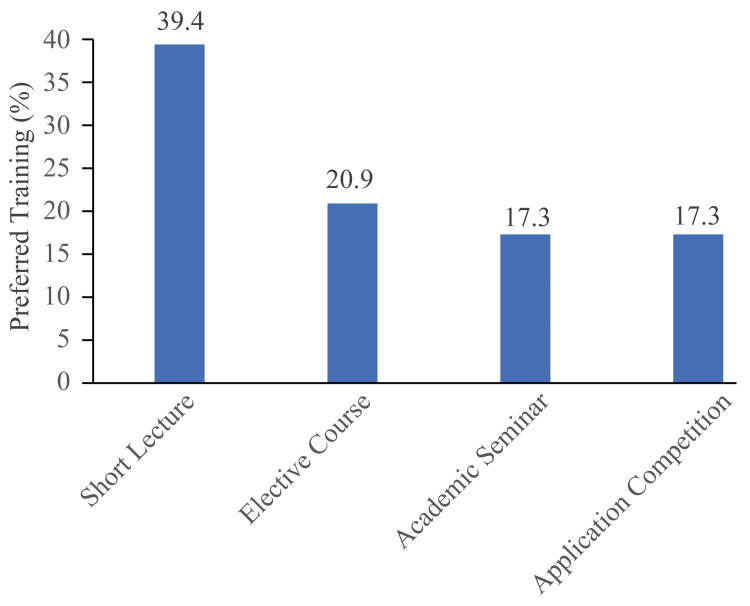
Medical Students' Preferred AILM Training format AILM: Artificial Intelligence Language Generation Model

In terms of improving training formats for AILM, the majority of students prefer short-term lectures (totalling three to five hours) (Figure 4).

Factors associated with opposition to the application of AILM in education

Among groups with supportive, neutral, and opposing attitudes towards the application of AILMs in medical education, univariate analysis showed that gender, specialty, academic performance, and prior experience with AILM were not associated with the attitudes to AILM in medical education. However, a higher proportion of those who opposed the use of AILM had advanced computer skills compared to those with basic computer skills (10/47, 13.5% vs. 9/177, 5.1%, P = 0.010). In contrast, there was no significant difference in computer skills between those with neutral and opposing attitudes (Table 1). To further explore the reasons for these differing attitudes, we conducted binary logistic regression analyses. First, we analyzed the factors associated with non-supportive attitudes (neutral and opposing attitudes) and found no significant independent predictors (Table 2).

**Table 2:** Factors Associated with Non-Supportive Attitudes Toward AILM Application AILM: Artificial Intelligence Language Generation Model

	Odds ratio	95% Confidence Interval	P value
Specialty			
Clinical	Reference		
Non-clinical	1.204	0.590 - 2.455	0.610
Academic performance			0.673
Top third	Reference		
Middle third	1.247	0.660 - 2.354	0.496
Bottom third	1.442	0.644 - 3.228	0.374
Computer skill			
Basic or intermediate	Reference		
Advanced	1.034	0.550 - 1.942	0.918

Next, we specifically examined the factors associated with opposition to AILM. After adjusting for specialty and academic performance in a multivariate logistic regression analysis, individuals with medium to high-level computer skills remained independently associated with opposition to the use of AILM, with an odds ratio of 2.959 (95% confidence interval 1.109 - 7.898) (Table 3).

**Table 3:** Factors Associated with Opposition to AILM Application AILM: Artificial Intelligence Language Generation Model

	Odds ratio	95% Confidence Interval	P value
Specialty			
Clinical	Reference		
Others	0.935	0.311 - 2.807	0.904
Academic performance			
Top third	Reference		
Middle third	1.236	0.417 - 3.662	0.702
Bottom third	1.449	0.393 - 5.347	0.577
Computer skill			
Basic or intermediate	Reference		
Advanced	2.851	1.068 - 7.609	0.036

## Discussion

This survey offers valuable insights into the utilization patterns and attitudes of Chinese medical students towards AILMs. Although many respondents have encountered these tools, their routine use is relatively rare. The moderate level of familiarity with the AILM indicates that while there is some recognition, there might be a need for increased exposure or education about their potential benefits. This is further supported by the fact that over half of the participants used AILMs less than once a week, and a small percentage (5.1%) had never used them at all. The majority of medical students support the integration of AILMs into medical education; however, concerns persist regarding the technological immaturity of AILMs, particularly the issue of “AI hallucination”, where the technology may generate plausible but inaccurate information.

Comparative insights with other nations

Our survey uncovered notable disparities in the awareness and utilization of AILMs among medical students in China compared to those in other nations. For instance, while nearly half of American medical students reported leveraging ChatGPT for writing tasks [7], Chinese students primarily used AILMs for more complex academic and professional challenges. This suggests that educational systems and cultural contexts may influence how AILMs are perceived and utilized. Beyond ChatGPT, locally developed AI solutions are gaining traction within the Chinese medical student community. Despite restricted access to ChatGPT and the abundance of domestic alternatives, respondents exhibited a preference for internationally renowned products like ChatGPT. This inclination suggests a prevailing belief in the superior quality or performance of these foreign offerings. Similar findings have been observed in other regions, yet with varying concerns and barriers to AI application. Veras et al. found that Canadian physiotherapy students have knowledge gaps regarding AI in their field and express higher concerns about the ethical implications of AI [15]. In contrast to the ethical concerns raised by Indian participants [12], our survey highlights concerns regarding the maturity of AILM technology. These insights underscore the urgency for enhancing the development of domestic AILM products, particularly in tailoring their knowledge bases to better suit the specific needs of medical education in China. In Turkey, the application of AI-generated radiological images in medical education has received positive feedback and improved learning outcomes. This comprehensive evaluation of the educational impact and effectiveness of specialized study modules in cross-sectional anatomy demonstrated that these modules significantly enhanced students' ability to identify anatomical structures in radiological images and distinguish sections from different levels and regions [16]. This highlights the potential benefits of integrating AI-generated content into medical curricula to improve educational outcomes.

Supportive view and nuanced perspectives

The mainstream supportive view for integrating AILMs into teaching, both currently (73.6%) and in the future (82.7%), demonstrates a positive outlook towards the role of AILMs in medical education. The significant correlation between these two attitudes suggests a consistent belief in their long-term potential. However, it is important to note that a notable portion of students (16.0%) hold neutral attitudes, which may stem from uncertainty about the benefits of AILMs, concerns about technological reliability, and a preference for traditional learning methods. 

The unexpected result that individuals with higher computer proficiency are less supportive of incorporating AILMs into medical education is inconsistent with the common belief that students with higher AI literacy would have greater support for the application of AI in medical education [11,17]. This discrepancy could be due to several reasons. Those with higher computer skills might have a more critical perspective due to their critical understanding of undeveloped technology of AILMs, including concerns about “AI hallucination”. It's possible they see limitations or flaws in AILMs that others might miss. Alternatively, they might feel more confident in their ability to solve problems without AILM assistance, reducing their perceived need for such tools. Additionally, the proficiency of AI in examinations raises concerns about its potential misuse for cheating [18]. This finding is further contextualized by a study on medical students' study preferences, which revealed that preferences for study methods and resources vary significantly across academic years and geographical regions [19]. For example, regular repetition was a predominant study method in certain regions, while others preferred correlating structures with functions or using mnemonics. These regional differences highlight the influence of educational contexts on students' preferences, suggesting that the reluctance to adopt new technologies like AILMs may be influenced by local educational practices and cultural contexts [19]. 

Another study among medical students found that individual learning styles significantly influence both study duration and theoretical course scores. Nearly half of the students preferred a reflective and theoretical approach to learning. Significant differences were observed in study duration and theoretical course scores, though not in gender. Understanding and accommodating these diverse learning styles can help optimize study duration and enhance academic achievement.

Future research should consider these factors to enhance the acceptance and effectiveness of AILMs in diverse educational settings.

Study limitations

This study has several limitations. The self-reported computer skills and academic performance by respondents may introduce subjective biases. While the anonymity of the survey likely encouraged honest responses, some respondents may still have provided socially desirable answers rather than their true opinions. Additionally, we did not include age in our analysis due to the minimal variation among our respondents, and this study was conducted only in the developed area of China. These factors limit the generalizability of our findings. In economically underdeveloped areas, due to limitations in educational resources, students have limited opportunities to access and use new technologies, and the promotion and application of AILMs will inevitably face more difficulties. The students may be more concerned about the cost of AILMs rather than the potential benefits, and they may pay less attention to issues such as data privacy, algorithmic bias, and medical decision-making responsibility. Thus, the feasibility of AILMs is somewhat overstated, if our results are extrapolated to underdeveloped areas. Future research should explore the specific applications and effectiveness of AILMs in medical education, particularly in economically underdeveloped regions [20]. Longitudinal studies tracking changes in students' attitudes over time are also recommended. Additionally, we did not conduct formal reliability testing (e.g., using Cronbach's alpha) for our questionnaire, which may limit the robustness of our findings. Lastly, the exclusion of responses with completion times less than 40 seconds, while intended to filter out insincere or careless answers, may not fully account for all such responses. To address these limitations, we ensured the robustness of our questionnaire through expert review and pilot testing, confirming content validity and question clarity. These measures may partially mitigate the limitations and enhance the overall quality and reliability of our findings.

Challenges and future directions

Despite the positive outlook towards AILMs, several challenges need to be addressed. Enhancing the quality and promotion of domestic AILM products is crucial. This involves refining technological capabilities and tailoring products to meet educational needs. Collaboration between educational institutions, developers, and healthcare professionals can ensure AILMs are both technologically advanced and pedagogically sound. Additionally, ethical and legal considerations, data privacy concerns, and the need for robust AI literacy among healthcare professionals are critical issues that must be addressed [20]. Further, the vast territory and economic diversity of China also need to be considered when interpreting the conclusions. During the period of this study, Deepseek had not yet entered the Chinese society. Since the main purpose of this survey is to analyze the foundation and attitude of medical students towards the application of AILMs, which is not closely related to specific AILM tools. However, with the rapid development and popularization of such emerging AILMs, the attitudes of medical students towards the application of AILMs may change. Therefore, the results of this study can only serve as a reference for the short-term introduction of AILMs. Future studies should employ mixed-methods approaches, combining questionnaires with in-depth interviews, to gain a more comprehensive understanding of students' attitudes towards AILMs. Longitudinal research tracking changes in attitudes over time is also recommended.

## Conclusions

In conclusion, while medical students generally exhibit openness towards the use of AILMs, several challenges need to be addressed to facilitate broader acceptance and integration. These challenges include improving and promoting domestic AILM products and understanding the diverse perspectives of individuals with varying levels of computer proficiency. Specific recommendations for policy and curriculum integration are to incorporate AILM training into medical school curricula, provide faculty development opportunities, allocate resources for AILM implementation, develop ethical guidelines, and establish assessment and feedback mechanisms. Implementing these recommendations can enhance the learning experience and outcomes for students.

## Appendices

Survey on the use of artificial intelligence language generation models among medical students

Section 1: Basic Information

1. Your gender (single choice):

A. Male

B. Female

2. Your undergraduate major is (single choice):

A. Clinical Medicine

B. Other medical fields

3. Based on your experience with computers,please select the option that best describes your current computer proficiency (single choice):

A. I cannot use a computer at all.

B. Basic use (e. g. ,browsing the web,sending emails,using social media)

C. Proficient in office software (e. g. ,Word,Excel,PowerPoint for document editing,data analysis,and presentation creation)

D. Intermediate software use (e. g. ,simple programming,image editing,audio/video playback and editing)

E. Advanced software use (e. g. ,advanced programming tools,complex database management,professional image and video editing software)

F. Professional-level skills (e. g. ,advanced programming,system development,network management,and security)

4. Your academic performance in your major (single choice):

A. Top third of the class

B. Middle third of the class

C. Bottom third of the class

Section 2: Awareness and Use of Artificial Intelligence Language Generation Models

5. Have you used ChatGPT? (single choice):

A. Yes

B. No

6. Besides ChatGPT, are you aware of any other Artificial Intelligence Language Generation Models? (multiple choice):

A. I am not aware of any.

B. iFLYTEK Spark Cognition Model

C. Tiangong AI

D. Wenxin Yiyan

E. Tongyi Qianwen

Other (please specify)

7. Among all the Artificial Intelligence Language Generation Models, which one would you prefer to use first? (single choice):

A. ChatGPT

B. Chinese-developed versions

8. Where did you learn about Artificial Intelligence Language Generation Models? (multiple choice):

A. Online media

B. Friends, family, classmates, or colleagues

C. Books

D. Lectures or conferences

E. Other (please specify)

9. How frequently do you use Artificial Intelligence Language Generation Models? (single choice):

A. Never

B. Occasionally, once a month

C. 1-2 times per week

D. 2-7 times per week

E. More than 7 times per week

10. In what situations do you most often use Artificial Intelligence Language Generation Models? (single choice):

A. Never used

B. No specific purpose, just out of curiosity

C. To solve daily life-related problems

D. To solve problems related to medical courses, professional studies, or academic issues

11. If you use Artificial Intelligence Language Generation Models for medical course learning, what purposes do you most commonly use them for? (multiple choice, up to 3 options):

A. To assist with learning, including searching for professional literature and creating study plans

B. For simulated skills training, including virtual patients and surgical simulations

C. To aid in clinical decision-making, including diagnostic and treatment suggestions

D. For data analysis, including clinical data management and epidemiological research

E. For research design and writing, including experimental design and paper writing

Section 3: Perceptions of Artificial Intelligence Language Generation Models

12. What do you think is the most prominent issue when using Artificial Intelligence Language Generation Models in medical course learning? (single choice):

A. The information provided by AI is incorrect or unavailable

B. Over-reliance on AI may reduce medical students' initiative to learn

C. The virtual interactive environment may lead to a decline in medical students' practical communication skills and hands-on abilities

D. Privacy and ethical issues

13. Do you think that Artificial Intelligence Language Generation Models will have a long-term impact on medical course learning for medical students? (single choice):

A. Unclear

B. Positive impact

C. Negative impact

D. Positive impact>Negative impact

E. Negative impact>Positive impact

14. Do you think there should be courses related to Artificial Intelligence Language Generation Models for medical students? (single choice):

A. Strongly agree

B. Agree

C. Neutral

D. Disagree

E. Strongly disagree

15. What do you think is the best format for teaching Artificial Intelligence Language Generation Models in medical schools? (multiple choice):

A. Short lectures (totaling 3-5 hours)

B. Elective courses

C. Competitions related to the application of Artificial Intelligence Language Generation Models

D. Academic seminars on Artificial Intelligence Language Generation Models

E. Other (please specify)
